# NGIWY-Amide: A Bioinspired Ultrashort Self-Assembled Peptide Gelator for Local Drug Delivery Applications

**DOI:** 10.3390/pharmaceutics14010133

**Published:** 2022-01-06

**Authors:** Nikoleta F. Theodoroula, Christina Karavasili, Manos C. Vlasiou, Alexandra Primikyri, Christia Nicolaou, Alexandra V. Chatzikonstantinou, Aikaterini-Theodora Chatzitaki, Christos Petrou, Nikolaos Bouropoulos, Constantinos K. Zacharis, Eleftheria Galatou, Yiannis Sarigiannis, Dimitrios G. Fatouros, Ioannis S. Vizirianakis

**Affiliations:** 1 Department of Molecular Pharmacology, School of Pharmacy, Aristotle University of Thessaloniki, 54124 Thessaloniki, Greece; theodorn@pharm.auth.gr; 2 Department of Pharmaceutical Technology, School of Pharmacy, Aristotle University of Thessaloniki, 54124 Thessaloniki, Greece; karavasc@pharm.auth.gr (C.K.); chatzita@pharm.auth.gr (A.-T.C.); dfatouro@pharm.auth.gr (D.G.F.); 3 Department of Life & Health Sciences, University of Nicosia, Nicosia 2417, Cyprus; vlasiou.m@unic.ac.cy (M.C.V.); krinikolaou@gmail.com (C.N.); petrou.c@unic.ac.cy (C.P.); galatou.e@unic.ac.cy (E.G.); 4 Department of Chemistry, University of Ioannina, 45110 Ioannina, Greece; a.primikyri@uoi.gr; 5 Biotechnology Laboratory, Department of Biological Applications and Technologies, University of Ioannina, 45110 Ioannina, Greece; achatzikonstantinou@uoi.gr; 6 Department of Materials Science, University of Patras, 26504 Patras, Greece; nbouro@upatras.gr; 7 Foundation for Research and Technology Hellas, Institute of Chemical Engineering and High Temperature Chemical Processes, 26504 Patras, Greece; 8 Laboratory of Pharmaceutical Analysis, Department of Pharmaceutical Technology, School of Pharmacy, Aristotle University of Thessaloniki, 54124 Thessaloniki, Greece; czacharis@pharm.auth.gr

**Keywords:** ultra-short peptides, smart materials, NGIWY-amide, self-assembled peptide hydrogels, drug delivery, sea cucumber

## Abstract

Fibrillar structures derived from plant or animal origin have long been a source of inspiration for the design of new biomaterials. The Asn-Gly-Ile-Trp-Tyr-NH_2_ (NGIWY-amide) pentapeptide, isolated from the sea cucumber *Apostichopus japonicus*, which spontaneously self-assembles in water to form hydrogel, pertains to this category. In this study, we evaluated this ultra-short cosmetic bioinspired peptide as vector for local drug delivery applications. Combining nuclear magnetic resonance, circular dichroism, infrared spectroscopy, X-ray diffraction, and rheological studies, the synthesized pentapeptide formed a stiff hydrogel with a high β-sheet content. Molecular dynamic simulations aligned well with scanning electron and atomic-force microscopy studies, revealing a highly filamentous structure with the fibers adopting a helical-twisted morphology. Model dye localization within the supramolecular hydrogel provided insights on the preferential distribution of hydrophobic and hydrophilic compounds in the hydrogel network. That was further depicted in the diffusion kinetics of drugs differing in their aqueous solubility and molecular weight, namely, doxorubicin hydrochloride, curcumin, and octreotide acetate, highlighting its versatility as a delivery vector of both hydrophobic and hydrophilic compounds of different molecular weight. Along with the observed cytocompatibility of the hydrogel, the NGIWY-amide pentapeptide may offer new approaches for cell growth, drug delivery, and 3D bioprinting tissue-engineering applications.

## 1. Introduction

Ultra-short peptide hydrogels, with two to seven amino acids, have gained the interest of scientists due to their involvement in important biological processes [1]. In addition to their advances in therapeutics [2], they are used in advanced nano-supramolecular technologies [3], novel smart bio-functional materials [4], vaccines [5], delivery systems for small drugs [6], biologics [7], and genes [8], as well as for cosmetics [9]. Furthermore, ultra-short peptide hydrogels, due to the ease of synthesis and scaling up, feasibility to modifications [10], non-immunogenicity, non-genotoxicity, biocompatibility and biodegradability, and mechanical stability [11], are widely used as starting materials for bioimaging probes [12], 3D bioprinting ink [13,14], and cell culture scaffolds for organoids [15]. Hydrogelation is mediated through hydrogen bonding, van der Waals forces, or π-π stacking of aromatic groups that drive the formation of nanofibrillar structures that further interweave into α-helices, β-sheets, hairpins, turns, micelles, ribbons, tapes, tubes, and coils [16,17]. Fibrillar structures are key elements in biological systems since they have been identified in both physiological (blood coagulation) and pathological conditions (interestingly, in degenerative diseases, e.g., Parkinson’s, Alzheimer’s) [18].

Drawing structures from nature to generate high-performance soft materials is a promising approach for the construction of functional biomaterials with advanced properties, like facilitating the transport processes inside organisms and molecular recognition. These moieties not only exhibit enhanced bioavailability but also act as “self-delivery” systems without involving other natural or artificial materials (hyaluronic acid, chitosan, etc). Moreover, light backbone and side-chain modifications of biomolecules in a cost-effective manner offer the essential fine-tuning in the structures to fabricate biofunctional materials with often-enticing chemical, physical, mechanical, and biological properties, ideal for a wide range of applications [19].

**Figure 1:**
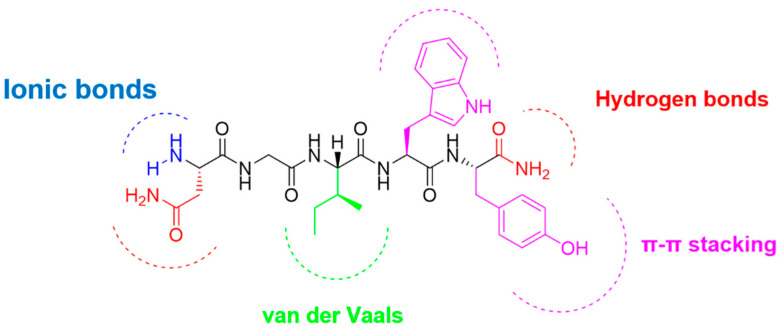
The structure of the pentapeptide NGIWY-amide.

NGIWY-amide (Asn-Gly-Ile-Trp-Tyr-NH_2_) (Figure 1) is among the few known neuropeptides isolated from echinoderms, in particular from the sea cucumber *Apostichopus japonicus* [20]. Localized in the holothurian nervous system, the peptide stiffens the dermis of the sea cucumber body wall. Dermis is a catch connective tissue, and its mechanical properties are modulated in response to other neuropeptides, like holokinins. Apart from its contractile action, the pentapeptide has been assessed for its gonadotropic action, as it induces oocyte maturation and gamete spawning of A. japonicus [21]. Compounds isolated from sea cucumbers are under extensive investigations applied in the cosmetic industry [22].

The balance between hydrophilicity and hydrophobicity can positively contribute to adequate gelation properties. The pentapeptide consists of two hydrophilic amino acids (N: Asn, G: Gly) at the N-terminal site and three hydrophobic amino acids at the C-terminal site, forming a small amphiphile. The backbone over-satisfies the criteria for gelling [10]. Naturally occurring aromatic amino acids Phe, Tyr, Trp, and even His form well-ordered nanostructures like fibrils, ribbons, or rods [23]. Two aromatic amino acids, tryptophan and tyrosine, are incorporated into the sequence and involved in intra- and intermolecular interactions due to the π-π stacking of the aromatic rings of the side chains. The backbone conformation of the peptide sequence is also responsible for its molecular self-assembly and mechanical stiffness. Glycine is among the amino acids that usually participate in the nanofiber’s formation. Isoleucine, an aliphatic amino acid, favors van der Waals forces and asparagine due to the side-chain carboxamide group inducing hydrogen bonds completing the most important interactions in peptide self-assembly. Hydrogen-bond formation, determining the secondary structure of the peptides, is also assisted by the C-terminal carboxamide. The peptide has a positive net charge of +1 due to the free amino group at the N-terminal site. The synergism and cooperativeness of all these weak interactions lead to a dynamic and responsive interplay around the peptide structure, leading to a functional material when it is exposed in an aqueous environment.

Yet, there is no study evaluating the ability of NGIWY-amide to self-assemble or its potential as a platform technology for local drug delivery or other similar applications. The self-assembling pentapeptide was studied in silico by using molecular dynamics (MD) simulations, and after the synthesis, the formed hydrogel was characterized and studied with nuclear magnetic resonance (NMR) for the inter- and intramolecular interactions of the peptide with the aqueous environment. Furthermore, an array of techniques, namely, atomic form microscopy (AFM) and transmission electron microscopy (TEM), Fourier transform–infrared spectroscopy (FT-IR) and X-ray diffraction (XRD), oscillatory rheology, UV/Vis, and fluorescence, were enrolled for the assessment of the peptide. Circular dichroism (CD) studies were used to obtain the essential information for the secondary structure of the peptide hydrogel. Confocal laser scanning microscopy (CLSM) was conducted for the localization of two model fluorescent dyes within the pentapeptide hydrogel. In vitro release studies and kinetic analysis were also performed, and cell culture-based biological assays were applied to certify the biocompatibility of the hydrogel.

## 2. Materials and Methods

### 2.1. Materials

Common solvents and reagents used in solid-phase peptide synthesis were purchased from commercial suppliers (Sigma-Aldrich, Merck–Millipore, etc.) and used without further purification unless otherwise noted. Rink Amide MBHA resin and Fmoc-protected amino acids were purchased from CBL Patras (Patras, Greece). ChemMatrix-Rink Amide resin was also purchased from Biotage (Uppsala, Sweden). Hydrochloric acid (HCl) (36.5–38%) was obtained from EM Industries, and acetic acid (AcOH) (99–100%) and ammonium hydroxide (NH_4_OH) (25%) were purchased from VWR Chemicals. Sodium deuteroxide (NaOD) (30% *w/v*, 99.5% D) and deuterium oxide (D_2_O) (99.9% D) were purchased from Cambridge Isotope Laboratories. Nile blue A, calcein, doxorubicin hydrochloride (98.0–102.0% HPLC), curcumin (from Curcuma longa), and trifluoroacetic acid (99%) were purchased from Sigma-Aldrich (Darmstadt, Germany). Acetonitrile (HPLC grade) was obtained from VWR Chemicals (Vienna, Austria). A B30 water purification system (Adrona SIA, Riga, Latvia) was utilized. Octreotide acetate was kindly gifted from Pharmathen (Athens, Greece). Milli-Q water was used in all studies.

### 2.2. Peptide Synthesis

The peptide was synthesized by using microwave-assisted solid-phase peptide synthesis on a single-channel microwave peptide synthesizer (Biotage^®^ Initiator+ SP Wave, Biotage, Sweden), standard Fmoc protection, and DIC/Oxyma pure activation strategies. Briefly, 0.3 g Rink Amide MBHA resin (100–200 mesh, 0.71 mmol g^−1^, CBL Patras, Greece) or H-Rink Amide ChemMatrix^®^ resin (100–200 mesh, 0.45 mmol g^−1^, Biotage, Sweden) were used to provide C-terminal peptide amides after the final cleavage from the solid support. The resin was swollen in N,N-dimethylformamide (DMF) at room temperature for 15 min under agitation (1200 rpm). The Fmoc protecting group was removed from the capped amino group by treatment of the resin with 2% DBU and 5% piperazine in DMF within 1 min at 90 °C under microwave irradiation. Couplings were performed under microwave irradiation for 5 min at 75 °C with 3.0 eq. of the required Fmoc amino acid, 3.3 eq. of N,N-diisopropylcarbodiimide (DIC), and 4.5 eq. of Oxyma pure as activator in DMF. After each deprotection step and coupling, a Kaiser test was performed to ensure completion of the reaction. After the completion of the synthesis, the resin was treated with a mixture of trifluoroacetic acid (TFA), triisopropylsilane (TIS), and water (95:2.5:2.5, *v/v*) (10 mL cleavage solution/g dry peptide–resin) at room temperature for 2.5 h to cleave the peptide from the resin as well as to remove the side-chain protection groups. The solution was filtered off and the solvent mixture was removed *under vacuo* and the white off peptide was recovered in chilled diethyl ether, then centrifuged to obtain the peptide precipitate, dissolved in water, and freeze-dried for 48 h. 

### 2.3. Characterization with Liquid Chromatography–Mass Spectroscopy (LC-MS) and Nuclear Magnetic Resonance (NMR)

The lyophilized crude peptide was analyzed for its purity (~92%) with high-performance liquid chromatography (Alliance HPLC e2695, PDA 2998, Waters, Milford, MA, USA) and electrospray ionization mass spectrometry (ACQUITY QDa Mass Detector, Waters, Milford, MA, USA). The peptide was eluted at 10.79 min through Hypersil Gold (100 × 2.1 mm, 3 μm) at a flow of 0.3 mL/min with a linear gradient system (5 to 100% B) for 30 min (A: 0.1% TFA in H_2_O, B: 0.1% TFA in MeCN (Figure S1)). The observed mass of the main peak corresponds to the [M + H]^+^ = 651.36 and the peak at [M + Na]^+^ = 673.45. ^1^H and ^13^C NMR spectra experiments were obtained in D_2_O on a Bruker AV500 spectrometer (Bruker Biospin, Rheinstetten, Germany) at 298 K using the Topspin 3.2 suite. Samples (0.5 mg) of the peptide were dissolved in 0.6 mL of deuterated water and transferred to 5 mm NMR tubes. Water suppression in the 1D ^1^H NMR and the 2D ^1^H-^1^H NOESY spectra was achieved using an excitation-sculpting pulse sequence. The mixing time in the 2D ^1^H-^1^H NOESY spectrum was set to 600 ms to obtain the maximum intensity. The 2D ^1^H-^13^C HSQC and ^1^H-^13^C HMBC NMR experiments were recorded using standard Bruker software (Table S1). 

The peptide hydrogel (1% *w/v*, 15.3 mM) was prepared in the tube (600 μL of D_2_O hot solution were transferred into the NMR tube and allowed to cool down and gelate inside the tube). By using excitation sculpting for water suppression (zgespg) with a recycle delay of 10 s, ^1^H NMR spectra were acquired. In the 2D ^1^H-^1^H NOESY spectrum, the mixing time of the hydrogel was set to 300 ms. Saturation transfer difference (STD) NMR experiments were performed with the selective saturation of a given ^1^H frequency by a train of 40 Gaussian pulses with a duration of 50 ms each, separated by a delay of 1 ms. The on-resonance irradiation was performed at a saturation frequency of −0.6 ppm, where only resonances of the hydrogel network can be encountered. Off-resonance irradiation was applied at 40 ppm, outside the spectra region of the ^1^H NMR resonances. The saturation time was set to 2 s. The STD spectrum was created by the subtraction of the on-resonance spectrum from the off-resonance spectrum.

### 2.4. Molecular Dynamics Simulations and Density Functional Theory Study

Avogadro software with the peptide-builder tool was used to create the peptide NGIWY peptide. Using the Assisted Model Building with Energy Recruitment (AMBER) 94 forcefield, a custom script was used to amidate the C-termini in VMD3. Proteins and nucleic acids were parameterized specifically. Only bonding and non-bonding terminology are used in AMBER, as well as a complex electrostatic treatment. For proteins, nucleic acids, and peptides, the results are excellent, but for other systems, such as bioinorganic and bioorganic systems, the results can be inconsistent. The simulations were run in NAMD 2.105 with final production trajectories of 200 ns. Scripts built in the Python6 programming language, as well as VMD, were used to process and further analyze trajectory data. The SURF computation (surface areas) was used to characterize the peptide structure, with the solvent probe radius set to 1.4 for any and all peptides. Clusters were established within 1.4 Å of each other in this way. The Kyte–Doolittle (KD) hydrophobicity indices were averaged over the amino acid sequence to obtain grand average hydropathicity (GRAVY) values. The optimized PM3 structures were used to distribute density functional theory calculations, with the ORCA program used to calculate the quantum mechanical descriptors associated with reactivity. DFT was used to perform geometry optimizations and frequency computations in the aqueous phase, utilizing the functional B3LYP, which could be a hybrid Hartree–Fock density functional theory (HF-DFT). 

### 2.5. Molecular Docking Study

The docking study was performed for the target peptide on the albumin using the AutoDock4 software. During this study, the crystallized protein located at the Protein Data Bank (PDB, https://www.rcsb.org/; last accessed 12 November 2021) was used. The water molecules co-crystallized with HSA structures were removed for the docking investigations, and the peptide recognition site was determined using residues within a grid of 60 Å × 60 Å × 60 Å, with a population of 100 randomly arranged individuals and a maximum number of 1.0 × 10^7^ energy assessments. Prior to docking, the target peptide was drawn within the Chem3D Pro and Chimera software, and the peptides were subjected to minimization energy using the hybrid functional B3LYP with a 6-311G (d, p) basis set. The conformation with the lowest free energy values of the peptide linked on HSA protein was used to calculate the ΔG (kcal/mol) of the peptide structure. Chimera software was used to create the figures for the protein and the peptide.

### 2.6. Peptide Supramolecular Hydrogel Formation and Characterization

To analyze the gelation behavior, gelation kinetics and the hydrogel’s viscoelastic properties were monitored on a Physica MCR 300 rheometer (Physica Messtechnic GmbH, Stuttgart, Germany) with cone-plate geometry (diameter 25 mm, cone angle 1°, gap 0.05 mm). The temperature was regulated at 25 ± 0.1 °C using a Paar Physica circulating bath and a controlled Peltier system (TEZ 150P/MCR). Oscillatory time-sweep experiments were performed at a frequency of 1 Hz and a strain of 0.1%, followed by frequency-sweep tests in the frequency range of 0.01–100 Hz and at a strain of 0.1%. Prior to the measurements, fresh peptide hydrogels were prepared in Milli-Q water at final concentrations of 2%. NGIWY-amide pentapeptide was dissolved in Milli-Q water, vortexed for 1 min, and further sonicated (Sonorex Digitec, Bandelin, Germany) at 25 °C for 10 min until a clear solution was obtained. 

FT-IR was performed to monitor the interactions in the amide I region (1550–1750 cm^−1^) using an IR Prestige-21 (Shimadzu). The FTIR spectrum in a wavenumber ranging between 4000 and 800 cm^−1^ was acquired in a sample of freeze-dried pentapeptide hydrogel (2% *w/v*). The crystallinity of the freeze-dried pentapeptide hydrogel was characterized via Bruker D8-Advance diffractometer with CuKα radiation (after gold coating) and the spectrum was recorded at 2θ from 10° to 60°. Freshly prepared peptide hydrogel samples (0.02% *w/v*) were used to record the UV-Vis and fluorescence absorbance spectra. The fluorescence emission spectrum (RF-5301 fluorophotometer, Shimadzu) was obtained over a range of 290–550 nm with excitation and emission slit widths set to 5 nm and 1.5 nm, respectively, and at an excitation of 280 nm. The UV-vis spectrum was recorded in the wavelength range of 200–800 nm on a UV-2501 spectrometer (Shimadzu). Circular dichroism experiments were carried out in double-distilled water using a Jasco J-1500 spectropolarimeter (Tokyo, Japan) equipped with a Peltier system for temperature control. Acquisitions were performed at 25 °C, between 190 and 260 nm with a 0.1 nm data pitch, 1 nm bandwidth, 100 nm/min scanning speed, and 1 s response time when a 0.1 cm quartz cuvette was used. When a 1 cm quartz cuvette was used, the acquisitions were performed at 25 °C between 190 nm and 260 nm with a 1 nm data pitch, 1 nm bandwidth, 500 nm/min scanning speed, and 1 s response time. All the spectra were acquired as an average of 3 scans and were corrected from a double-distilled water reference solution. The results were analyzed using Jasco Spectra Manager software.

### 2.7. Morphological Assessment

The hydrogel microstructure was visualized with atomic form microscopy (AFM) using a Veeco Multimode AFM (Veeco Instruments, Inc., Santa Barbara, CA, USA) with a Nanoscope IIIa controller. A 10 μL drop of the pentapeptide hydrogel (2% *w/v*) was transferred by pipetting onto a mica surface. After 30 s, the mica was rinsed with 300 μL Milli-Q water and air-dried. The height images were acquired using cantilevers with a spring constant of 10 N/m while the scan rate was adjusted to 1 Hz. 

For transmission electron microscopy (TEM) visualization of the peptide hydrogel (2% *w/v*), an aliquot of 10 μL was deposited on carbon film-coated grids and air-dried prior imaging with TEM Jeol 2100 operated at 200 kV. ImageJ software was used to determine the mean fiber diameter by randomly measuring 100 fibers from the TEM images.

### 2.8. Confocal Laser-Scanning Microscopy (CLSM) and In Vitro Drug Release Studies and Kinetics

Localization of two model fluorescent dyes, namely, Nile blue (lipophilic) and calcein (hydrophilic), within the pentapeptide hydrogel microstructure was assessed with confocal laser-scanning microscopy. Two microliters of Nile blue (100 μg/mL in ethanol) or calcein (400 μg/mL in water) were loaded into 20 μL of the peptide hydrogel (2% *w/v*). Samples were imaged with a 63× oil-immersion lens under a Zeiss LSM 780 CLSM (Carl Zeiss Microscopy GmbH, Berlin, Germany) using appropriate filters. Images were obtained using ZEN 2011 software.

The in vitro release profiles of octreotide acetate, doxorubicin hydrochloride, and curcumin from the pentapeptide hydrogel (2% *w/v*) were recorded in phosphate-buffered saline (PBS, pH 7.4) at 37 °C, whereas in the case of curcumin, Tween 80 (0.1% *v/v*) was added in the medium to assure sink conditions. The drug-loaded pentapeptide hydrogels were prepared at a final drug concentration of 0.5 mg/mL for octreotide acetate and doxorubicin hydrochloride and at 0.2 mg/mL for curcumin. Release studies were conducted in Eppendorf tubes, in which the drug-loaded hydrogels (50 μL) were left overnight at RT, prior to the gentle addition of 1 mL release medium on top of them. At predetermined time-points, samples (800 μL) were collected and replaced by an equal amount of fresh and prewarmed medium. HPLC analysis was performed for the quantification of octreotide acetate, whereas doxorubicin and curcumin were quantified by fluorescence spectroscopy. Excitation and emission wavelengths were set to 488 nm and 590 nm, respectively, for doxorubicin hydrochloride (excitation/emission slit widths: 5 nm/10 nm) and at 420 nm and 550 nm for curcumin (excitation/emission slit widths: 5 nm/10 nm). The release kinetics of all drugs from the peptide hydrogel were analyzed according to different mathematical models (zero order, first order, Korsmeyer–Peppas, Higuchi, Hixon–Crowell, and Weibull) using the excel add-in software DDSolver. For the analysis of octreotide acetate, an HPLC method was developed and validated in-house. HPLC instrumentation (Shimadzu, Kyoto, Japan) consisted of two LC-20AD isocratic high-performance pumps, an autosampler (SIL-20C HT), a column oven (CTO-20AC), and a photo-diode array detector (SPD-M20A). LabSolutions software (vs. 5.42SP3) was utilized for HPLC instrument control and operation. The separation of octreotide from the sample matrix was performed using a Nucleodur C18 analytical column (125 × 4.6 mm, 5 μm) (Macherey-Nagel, Germany). The column oven was set to 30 °C. The mobile phases were water (mobile phase A) and acetonitrile (mobile phase B), both containing 0.1% *v/v* trifluoroacetic acid. A binary gradient elution was used, starting from 5% *v/v* B and followed by a linear increase to 50% *v/v* B in 6 min. Then it was kept constant for up to one minute and altered to 5% B at 8 min. Then, the column was equilibrated for 15 min (at the initial conditions) in order to obtain reproducible retention times. A volume of 20 μL was injected to the column while the flow rate was set to 1 mL min^−1^. The octreotide was monitored at 210 nm. The processed samples were kept at 10 °C in the autosampler tray prior to analysis. A washing mixture of 50/50% *v/v* H_2_O/CH_3_OH was employed between analyses in order to avoid potential carryover. 

Using the above conditions, the octreotide peak was well resolved from the matrix, with a retention time of 11 min. The linearity of the HPLC method was investigated in the range of 0.5–50 μg/mL with R^2^ = 0.9982 using 7 calibrants prepared in water. The limit of quantitation (LOQ) was 0.5 μg mL^−1^ and the % RSD was less than 1.0% (*n* = 3).

### 2.9. In Vitro Cell Viability and Cell Death Assays

The cell lines MRC5 (normal) and HSC3 (malignant) were grown in culture in Dulbecco’s Modified Eagle Medium (DMEM) supplemented with 10% (*v/v*) fetal bovine serum (FBS) and containing 1% *v/v* penicillin–streptomycin solution. The incubation of cells was at 37 °C in a humidified atmosphere with 5% *v/v* CO_2_. Before each experiment, the cells were washed with phosphate-buffered saline (PBS) pH 7.4 and then were harvested by trypsinization and centrifuged at 1000 rpm for 5 min. After resuspension, 5 × 10^3^ cells were seeded per well in a 96-well plate and incubated as mentioned above. The following day, various amounts of the NGIWY amide peptide (0, 0.01, 0.1, 0.2, 0.4, 0.6 mg/mL) were separately added in cultures to assess cell viability. Five independent biological experiments were conducted for the measurement of cell viability for each concentration and all data presented are the average from triplicate experiments. After 24, 48, and 72 h of incubation. Cell Counting Kit-8 (CCK8, St. Louis, MO, Sigma-Aldrich) reagent was added to each well, and after incubation for 2 h at 37 °C, the OD was assessed at 450 nm in a multifunction microplate reader. Wells containing only the CCK-8 reagent were used as blank control.

Complementary to cell viability assessment, the number of dead cells in cultures exposed to various concentrations of the NGIWY amide peptide (0, 0.01, 0.1, 0.2, 0.4, 0.6 mg/mL) mentioned above was also determined using the trypan blue exclusion method, as previously described [24].

### 2.10. Statistical Analysis

Data are presented as mean ± standard deviation (SD) of triplicate incubations. Statistical analysis was performed using one-way analysis of variance (ANOVA) and significance level was set at *p* < 0.05.

## 3. Results

### 3.1. Synthesis and Characterization of the Self-Assembled Pentapeptide

NGIWY-amide high-performance liquid chromatography and electrospray ionization mass spectrometry (LC-PDA-ESI-MS) revealed the high purity of the peptide and the expected molecular mass of 650.36, as shown in Figures S1 and S2, respectively. Analytical data of the NMR experiments, ^1^H and ^13^C NMR spectra obtained in D_2_O, are displayed in Table S1 of the Supporting Information. 

### 3.2. NMR Studies 

#### 3.2.1. 1H NMR of the Peptide Hydrogel

**Figure 2:**
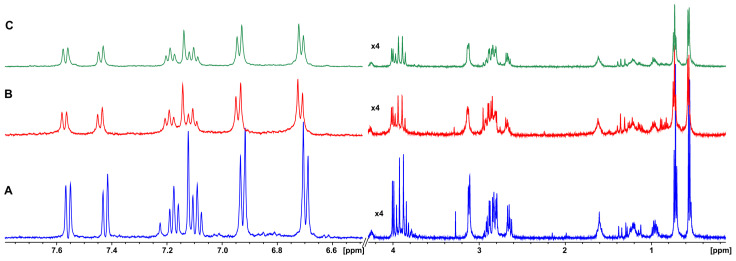
(**A**) ^1^H NMR spectrum of NGIWY (1.3 mM) peptide in solution (500 MHz, PBS in D_2_O pH 7.4). (**B**) ^1^H NMR spectrum of NGIWY (1% *w/v*, 15.3 mM) (500 MHz, PBS in D_2_O pH 7.4) ~10 min after the initial preparation. (**C**) ^1^H NMR spectrum of NGIWY (1% *w/v*, 15.3 mM) (500 MHz, PBS in D_2_O pH 7.4) 24 h after the initial preparation.

The extent of the gelator molecule incorporation into the hydrogel network was assessed with 1D ^1^H NMR spectroscopy, since these molecules do not become observable with solution-state NMR spectroscopy. Changes in the peak intensities, linewidths, and chemical shifts allow for the evaluation of the ratio between the free molecules in the isotropic phase and the molecules forming the hydrogel fibers [25]. All sharp and intense ^1^H NMR peaks of the NGIWY peptide in solution were broader and less intense in the ^1^H NMR spectrum of the NGIWY hydrogel since all the peaks of the peptide reflected the state in the hydrogel form (Figure 2A,B). The linewidth was further increased, and broadening reached a plateau 24,332 h after the gel preparation (Figure 2C). Approximately 50% of gelator molecules became structural components of the hydrogel fibers. However, the exact percentage of the incorporation is highly dependent on the time of the acquisition of the initial spectrum and on the kinetics of the gelation due to factors such as temperature.

#### 3.2.2. NOESY NMR of the Peptide Hydrogel

**Figure 3:**
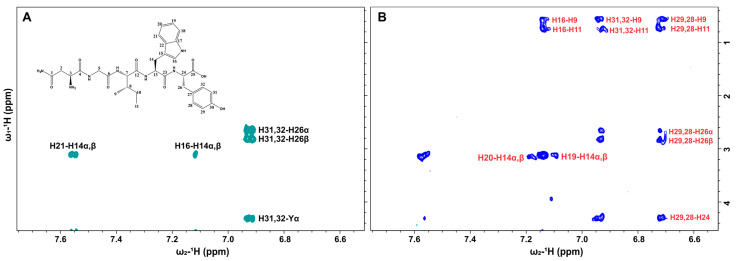
(**A**) Selective region of 2D NOESY NMR spectrum of NGIWY peptide (1.3 mM) in solution (500 MHz, PBS in D_2_O pH 7.4). (**B**) Selective region of 2D NOESY NMR spectrum of NGIWY peptide (1% *w/v*, 15.3 mM) (500 MHz, PBS in D_2_O pH 7.4). New Tr-NOE cross-peaks are denoted with the red color.

A ^1^H–^1^H NOESY (nuclear Overhauser effect spectroscopy) spectrum was recorded for the NGIWY peptide in the soluble form at a concentration of 1.3 × 10^−3^ M. The NOESY spectrum showed several well-resolved weak positive NOEs (cross-peaks with opposite sign with respect to the diagonal), as expected for a low-molecular-weight molecule (Figure 3). The intermolecular interactions responsible for the formation of the hydrogel network were then investigated and in the 2D NOESY spectrum of the hydrogel, strong negative NOE enhancements appeared. This effect is characteristic for large-molecular-weight molecules that transfer magnetization efficiently through dipolar interactions. Due to fast dynamics of exchange between solution and gel states in the NMR frequency time scale, information was transferred from the hydrogel fibers to the molecules in solution, resulting in strong negative NOEs on the 2D map [26]. Interestingly, several new NOEs appeared in the spectrum of the hydrogel, including cross-peaks between the methyl protons 9 and 11 of Ile and all the aromatic protons of (i) Tyr (H28, H29, H31 and H32) and (ii) H16 of Trp, suggesting a possible folding of the two aromatic regions of Tyr and Trp resulting in a closer proximity to Ile. In addition, protons H9 showed new NOEs with proton H10β of Ile and the aromatic protons H28 and H29 of Tyr with H26α, H26β, and H24 protons of the same residue. Protons H19 and H20 demonstrated weak negative NOEs with proton H14α,β of Trp, indicating spatial proximity. Additionally, the NOEs between protons H8 and H9 and protons H10α and H10β of Ile remained as weak positive NOEs, which was expected since they are attributed to a TOCSY effect and spin-spin diffusion occurs through bonds.

#### 3.2.3. Saturation Transfer Difference (STD) NMR

**Figure 4:**
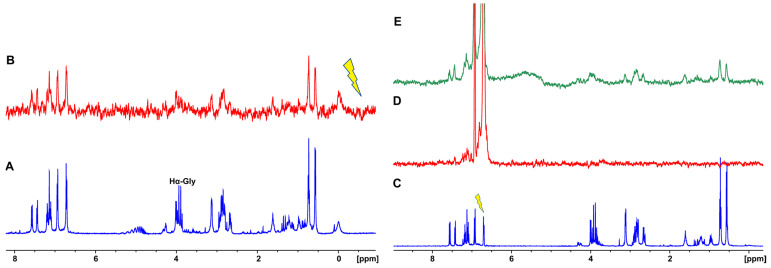
(**A**) ^1^H NMR spectrum of NGIWY (500 MHz, PBS in D_2_O pH 7.4). (**B**) STD NMR spectrum of NGIWY (1% *w/v*, 15.3 mM) peptide (500 MHz, PBS in D_2_O pH 7.4). On-resonance selective irradiation occurred at −0.6 ppm. (**C**) ^1^H NMR spectrum of NGIWY (1% *w/v*, 15.3 mM) (500 MHz, PBS in D_2_O pH 7.4). (**D**) STD NMR spectrum of NGIWY peptide (1.3 mM) in solution (500 MHz, PBS in D_2_O pH 7.4). On-resonance selective irradiation occurred at 6.70 ppm. (**E**) STD NMR spectrum of NGIWY (1% *w/v*, 15.3 mM) (500 MHz, PBS in D_2_O pH 7.4). On-resonance selective irradiation occurred at 6.70 ppm.

Further investigation of the exchange phenomena between the molecules in the solution state and the molecules incorporated into the hydrogel fiber network was performed with saturation transfer difference (STD) NMR experiments. STD NMR experiments are based on the nuclear Overhauser effect and on the magnetization transfer via spin diffusion from a selectively irradiated macromolecular receptor to the protons of a ligand that are in close contact with the receptor (≤5 Å) [27]. The applications of STD NMR spectroscopy in the study of supramolecular gels and amino acid-based hydrogels have been investigated very recently [26]. In this case, the hydrogel fibrous network can be considered the macromolecular receptor that is selectively saturated. The selective saturation of the hydrogel can be transferred through the nuclear Overhauser effect throughout the network, resulting in the bound gelator molecules, which are then dissociated back to the pools of water transferring the saturation in the isotropic solution phase. Only the STD signals of the gelator molecules that have been in contact with the hydrogel fibers and have received magnetization transfer will appear in the spectrum. We selectively saturated a region below 0 ppm where only resonances of the network were present and observed STD signals for all the amino acid gelator molecules that received saturation from the fibrous network. The STD signal of Gly H5 was significantly lower compared to the rest of the molecules, possibly indicating a strong binding with longer residence time in the hydrogel network resulting in lower magnetization transfer and less intense STD signals (Figure 4A,B) [25]. We then investigated the magnetization transfer occurring upon the selective irradiation of the H28 and H29 aromatic protons of Tyr residue (Figure 4C–E). In the case of the NGIWY peptide in solution (1.3 mM), except for the nearby H31, H32, H19, H16, and H_2_0 protons, which are within the 0.5 ppm regime of irradiation specificity, no other STD signals appeared in the STD spectrum, suggesting that magnetization was not transferred to the rest of the protons (Figure 4D). On the other hand, when the Tyr aromatic protons H28 and H29 were selectively irradiated in the case of the hydrogel, the STD signals of all the amino acids appeared in the spectrum (Figure 4E). This could be explained by the fact that magnetization was transferred from the gelator molecules to the hydrogel network and from the fibers back to all the protons of the gelators. Again, the STD signal of Gly H5 was lower due to stronger binding.

### 3.3. Molecular Modelling

#### 3.3.1. Molecular Dynamics Simulations

Molecular dynamics (MD) simulations are used to investigate the atomically detailed molecular interactions underlying peptide self-assembly processes. MD simulations provide a reliable method for examining the structural characteristics and conformational dynamics of designed peptides, and they may provide experimentally unavailable insight into the dynamical foundation of self-assembly. We used MD simulations in this study to evaluate the formation of structural characteristics in a peptide system and provide an atomistic view of the nanostructure’s self-assembly process. We conducted MD simulations of an empirically confirmed pentapeptide (NGIWY) sequence that may assemble in aqueous conditions to explain the molecular-scale mechanisms related to self-assembly. These extended (200 ns), all-atom simulations were performed in explicit solvent using the AMBER 94 force field. As detailed in several primers, such forcefields represent the physicochemical properties of each amino acid, including partial charges, atomic interaction potentials, and other factors, using a classical, molecular mechanics-based method. In fact, AMBER 94 could be a cutting-edge force field that is applied to a variety of biomolecular systems, particularly peptides [28,29].

**Figure 5:**
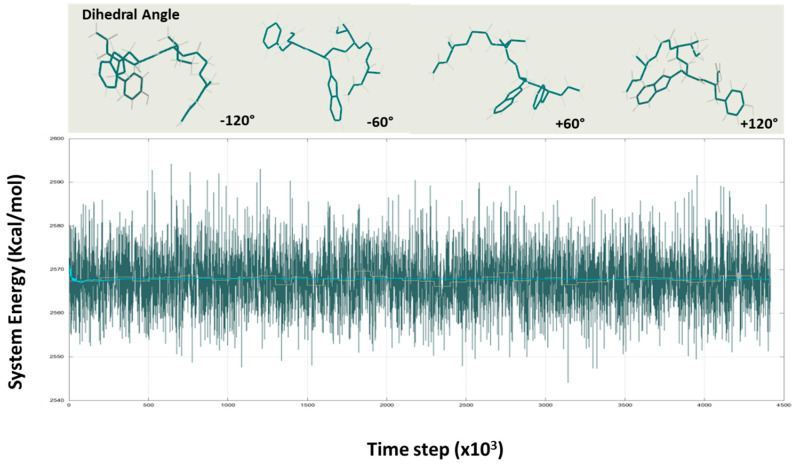
Dihedral angle conformations of the NGIWY peptide and energy formation during the simulation process.

The assembly propensity of short peptides correlated with the diffusional association of the NGIWY peptide, according to our models. The peptides took on a variety of aberrant conformations, with transiently stable turns “flickering” into existence. Individuals could be identified in less than 50 ns. NGIWY peptide nanofibers assembled into a β-sheet secondary structure. Extending the simulation further yielded peptides that assembled into two large clusters of tight turns and a random coil by ≈200 ns, indicating a structure between the random coil and β-sheet. We used a discrete number-density function to quantify the aggregation propensity of the peptide along its respective trajectories. For NGIWY, a detectable, and presumably hydrophilic-driven, “collapse” of the system appeared to be more kinetically allowed versus other sequences. Visual inspection of trajectories showed a pointy structural reorganization that was timely (<100 ns) in most of the simulations (Figure 5). This random coil formation suggests that the NGIWY peptide is dynamic and that its conformation adapts to its environment. The tight turn depicted in Figure 5 is attributed to the calculated β-turn of the peptide. 

Because the interactions involved in turn formation are mostly local, turns have been argued to be significant in folding because they can commence productive structure creation without a large loss in chain entropy [28]. With the exception of the N- and C-termini, the torsion angles for amino acids suggest significant structural heterogeneity in the system. Our findings imply that the pentapeptide’s central Ile frequently adopted a type-II turn conformation (φ = −60°, φ = 120°) or an antiparallel sheet structure (φ = −120°, φ = 60°), (Figure 4). B-turns are well suited to participating in ligand binding, molecular recognition, protein–protein, or protein–nucleic acid interactions because they are largely surface-exposed, influencing protein activities and intermolecular interactions. Because the interactions involved in turn formation are mostly local, turns have been argued to be significant in folding because they can commence productive structure creation without a large loss in chain entropy [30].

#### 3.3.2. DFT Study

**Figure 6:**
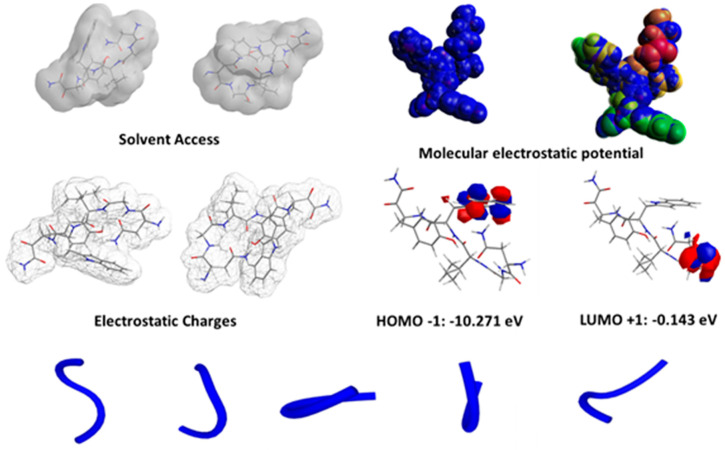
Information received by the DFT studies revealed a fully hydrophilic peptide and an electrostatic potential profile. The MD simulations showed the 5 most possible conformations of the pentapeptide.

The molecular characteristics of the octanol–water partition coefficient (logP), the Δ_GAP_ energy (Δ_GapE_), and hence the potential of ionization (I), were all indirectly related to biological activity, according to the models. However, the energy of the HOMO orbital (E_HOMO_), overall absolute charge, and Ghose–Crippen molar refractivity (AMR) are descriptors that were directly associated with the activity and affinity of the peptide. This was likely due to the peptides’ electron-rich side-chain residues, which were related to high HOMO properties (Figure 6).

ADME models are used to describe biological activity. The thermodynamic and transport properties of the molecular system in Table S2 lists the peptide’s molecular properties, and Table S3 shows the values of the topological and molecular descriptors that were used in the simulation algorithm study. The van der Waals attractions between molecules play a role in determining the thermodynamic and transport properties of a chemical system. The electronic polarizability and molar refractivity (AMR) of molecules are linked by van der Waals interactions (or London scattering attractions) (Figure 6). AMR is a constitutive additive feature that can be estimated to account for contributions from within the atom and its connectivity, as well as a variety of correction variables [31,32]. 

AMR could be a crucial physical–chemical parameter that has contributed significantly to the study of binding electrons in organic compounds in the past. The results of the AMR and charge values show that this ultra-short peptide is comparable to other short peptides from the literature. Furthermore, the descriptors associated with the peptide’s water solubility indicate that its activity is linked to its ability to be water soluble, and NGIWY is a very soluble peptide. However, peptides with higher lipophilicity do not present higher biological activity [33].

Quantum mechanical descriptors generated from DFT investigations have been used successfully to justify and explain chemical reactivity in a wide range of applications. One of the most widely used quantum mechanical descriptors is the energy of the highest occupied molecular orbital (EHOMO). The frontier orbitals and their energies are significant in explaining a system’s reactivity and are utilized as a marker of high-electron-density locations (Figure 6). As a result, these zones exhibit a positive region to be attacked by electrophile groups.

#### 3.3.3. Binding Free Energy Calculation

The protein–protein docking analysis employed MD simulations to find the most crowded peptide configurations, allowing for the determination of protein sites involved in the recognition process (Figure 5). Docking experiments, on the other hand, do not demonstrate the validity of those interactions in a highly solvated and varied environment. Because of the initial configurations for the 20 ns-long MD simulation, complexes with the lowest binding free energy generated by docking calculations were frequently chosen. The binding free energy (ΔGbind) without the entropic contribution (ΔTS) was energetically beneficial for the complex, according to this research. However, docking studies do not illustrate the soundness of those interactions in an exceedingly solvated and versatile environment. The electrostatic energy (ΔEelec) and hence the solvation energy (ΔG_GB_) varied more than the van der Waals dispersion energies (Gvdw), as seen in Table S4. The non-polar contributions (ΔEnon-polar = ΔEvdw + ΔG_SA_) dominated the net ΔG bind value. The screening effect of the solvent is evident here, with non-polar contributions dominating the total ΔGbind value. These results reveal that the NGIWY peptide experienced the foremost favorable binding free energy with the has receptor, compared to other short peptides from the literature, indicating a great binding result with the transport protein, a promising result for drug delivery systems [34,35]. Structural analysis of the HSA–peptide complex shows that it maintained similar structural conformation together with MD simulations, like those predicted through docking procedures.

### 3.4. Pentapeptide Hydrogel Preparation and Characterization

#### 3.4.1. Hydrogel Preparation and Rheological Studies

NGIWY-amide pentapeptide was dissolved in Milli-Q water at a 2% (*w/v*) final concentration and sonicated at 25 °C until a clear solution was obtained. The solution was left undisturbed, and a transparent gel was obtained within less than 30 min (Figure S3).

**Figure 7:**
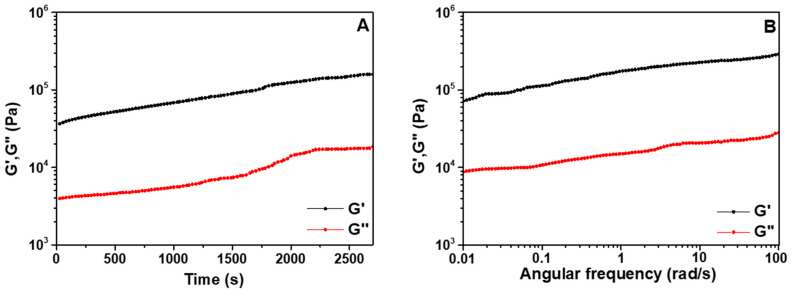
Oscillatory (**A**) time-sweep and (**B**) frequency-sweep measurements of the pentapeptide hydrogel (2% *w/v*) at a constant frequency of 1 Hz and strain of 0.1% performed at 25 °C.

The gelation kinetics and viscoelastic properties of the pentapeptide hydrogel were assessed with rheological analysis. As seen in Figure 7A, the elastic modulus G’ was an order of magnitude higher than the loss modulus G’’ already from the beginning of the gel-curing experiment, indicating the formation of a firm hydrogel early upon hydration of the pentapeptide. A minor increase in G’ at ca. 28 min, observed as change in the slope, denotes the pentapeptide hydrogel’s onset of gelation, which continued to stiffen with time (G’max: 160 kPa). Effective gelation depends on the presence of a hydrophobic/hydrophilic balance in the amino acid sequence of a peptide [36,37], in which the presence of aromatic groups has been found to be a facilitator of the gelation process [38]. This observation further supports the experimental findings relative to the pentapeptide sequence containing amino acids (tyrosine and tryptophan) with aromatic moieties. The viscoelastic properties of the pentapeptide hydrogel were monitored immediately after the end of the gel-curing experiments in the frequency range of 0.01–100 rad/s and at 0.1% strain (Figure 7B). The dominancy of the elastic response of the pentapeptide hydrogel was evident over the frequency range tested. The storage modulus G’ was considerably higher than the loss modulus G’’, indicating the formation of a stiff and stable hydrogel network.

#### 3.4.2. Optical Properties, Structural Conformation, and Crystallinity of the Pentapeptide Hydrogel

**Figure 8:**
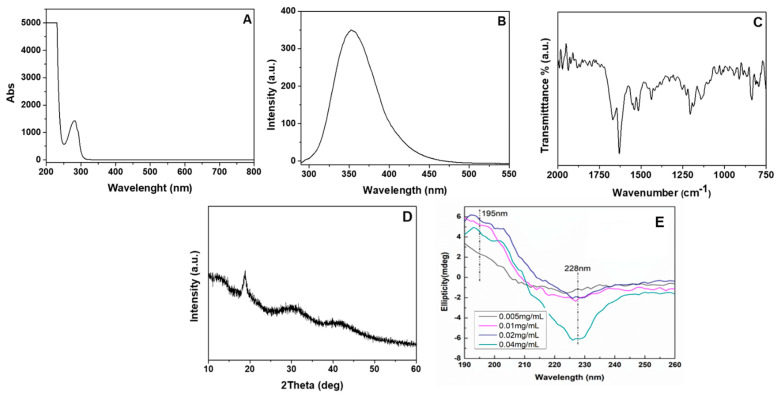
(**A**) UV adsorption and (**B**) fluorescence spectra of the pentapeptide hydrogel (0.02% *w/v*) in water. (**C**) FTIR and (**D**) XRD diffractogram of the pentapeptide hydrogel. (**E**) CD spectra of the pentapeptide at different concentrations in double-distilled water at 25 °C.

The light-absorption properties of the pentapeptide hydrogel were investigated by recording its UV-Vis spectrum in aqueous medium (Figure 8A). The most characteristic absorption features of the pentapeptide were observed in the far-UV (<220 nm) and the near-UV regions (280 nm). In particular, the strong absorption peak observed below 220 nm is attributed to the π
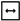
π* transitions of the peptide bonds [39,40] and the carboxylic acid moieties in the peptide [41,42]. The absorption peak at 280 nm is attributed to the aromatic side chains of Trp, with a less intense π
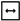
π* transition identified as a shoulder at 292 nm. Even though less intense, the absorption of Tyr significantly overlapped with that of Trp in the same region. The fluorescence properties of the pentapeptide hydrogel were assessed after dilution of the hydrogel with Milli-Q water at a final concentration of 0.02% *w/v*. The intrinsic fluorescence of the pentapeptide derived from the excitation of Trp and Tyr, with Trp being significantly more fluorescent than Tyr [39]. As seen in Figure 7B, the pentapeptide exhibited a strong emission peak at 354 nm, due to the presence of the indolic ring of Trp. On the other hand, the fluorescence of tyrosine, due to the presence of its phenolic group, was observed as a shoulder at 295 nm [43], since its emission fluorescence was quenched by the presence of the adjacent tryptophan via resonance energy transfer and through ionization of its aromatic hydroxyl group.

FT-IR spectroscopy was conducted to evaluate the secondary conformation of the pentapeptide hydrogel (Figure 8C) [44]. The amide I strong absorption band observed at 1629 cm^−1^ is attributed to the stretching vibrations of the C=O and C-N groups [45] and indicates the occurrence of a high β-sheet structure content [46], whereas the peak present at 1668 cm^−1^ suggests the presence of β-turns. Beta sheets that obtain an antiparallel conformation typically demonstrate a weak band at ca. 1690 cm^−1^ [25]. The presence of the high-intensity amide I band at 1629 cm^−1^ and the absence of a weak band at 1690 cm^−1^ suggest the formation of a parallel β-sheet conformation in the pentapeptide hydrogel [27]. Amide II bands observed at 1541 cm^−1^ and 1516 cm^−1^ are associated with N-H bending vibration and the C-N stretching vibration. XRD analysis was conducted to gain further information on the peptide molecular packing in the hydrogel (Figure 8D). The freeze-dried pentapeptide hydrogel demonstrated a peak at a d-spacing of 4.7 Å (2θ = 18.79°), attributed to the interchain distance expected for β-sheet structures [47]. CD spectroscopy was also employed for the evaluation of the secondary conformation of the pentapeptide at different concentrations (Figure 8E). A positive broad peak around 195–200 nm along with a negative peak at 226–229 nm indicate the presence of a β-sheet like arrangement. The red-shifted β-sheet signal has been previously observed and it has been shown to correlate with a twisted structure [48]. Additionally, the changes observed in the CD spectra as a function of the change in sample concentration are a feature of a self-assembly process that takes place in the solution [49]. 

#### 3.4.3. Morphological Evaluation of the Pentapeptide Hydrogel

**Figure 9:**
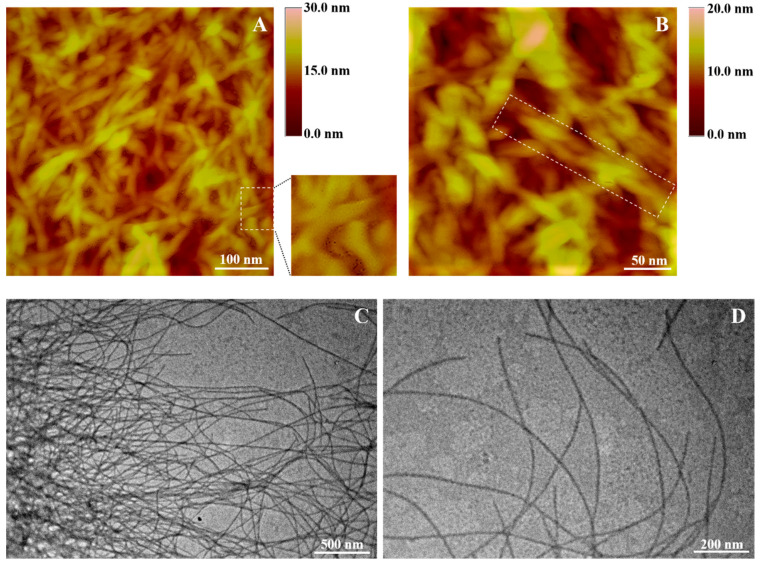
(**A**,**B**) AFM images and (**C**,**D**) TEM micrographs of the NGIWY-amide pentapeptide hydrogel prepared at 2% *w/v* in Milli-Q water.

The AFM images of the pentapeptide hydrogel at the concentration of 2% *w/v* demonstrated a surface topology of a network of intertwined nanofibers with helical-twisted morphology (Figure 9A,B dotted rectangles). TEM analysis further verified the findings of AFM studies, showing that the NGIWY-amide pentapeptide was highly filamentous, forming micrometer-long fibers with twisted morphology and a mean diameter of 5.6 ± 1.1 nm. It has been previously shown that a short biomimetic eight amino acid residue peptide derived from squid-sucker ring-teeth proteins gels in water, forming a network of twisted nanofibers with a width of less than 10 nm and a periodicity of 80 nm along the fibers [50]. Experimental findings based on both physicochemical studies and image analysis align well with MD simulations on the possible conformation adopted by the NGIWY-amide pentapeptide, amply indicating for the adoption of a β-turn/β-sheet secondary structure.

### 3.5. Model Dye Localization in the Hydrogel Network and In Vitro Drug Release Studies 

Aiming to identify the suitability of the NGIWY-amide pentapeptide hydrogel as a carrier of both hydrophobic and hydrophilic compounds, the distribution of a lipophilic and a hydrophilic fluorescent dye within the peptide hydrogel was initially evaluated and visualized with CLSM (Figure S4). A strong blue fluorescent signal delineating the peptide nanofiber was evidenced in the presence of Nile blue within the pentapeptide hydrogel, indicating the preferable distribution and interaction of the lipophilic dye with the hydrophobic domains of the pentapeptide. This could translate to a slower dissociation of the lipophilic compound from the nanofibers and a more retarded diffusion towards the aqueous matrix of the hydrogel and further to the surrounding medium. On the contrary, the more hydrophilic calcein demonstrated a more scattered fluorescent signal, suggesting a more preferential distribution within the aqueous environment of the hydrogel rather than within the peptide nanofibers. 

**Figure 10:**
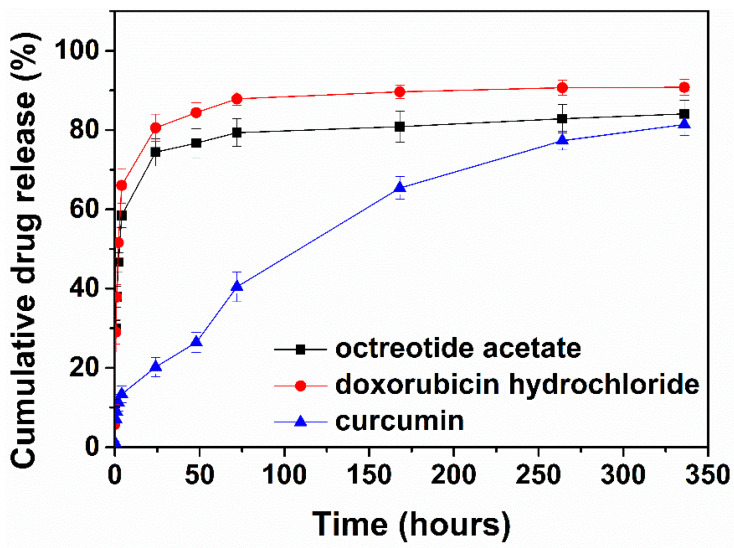
In vitro release profiles of octreotide acetate, doxorubicin hydrochloride, and curcumin from the pentapeptide in PBS pH 7.4 (in the presence of Tween 80 0.1% for curcumin) at 37 °C (*n* = 3, ±S.D.).

In order to verify the CLSM finding, three drugs differing in their molecular weight and aqueous solubility were selected and their in vitro release profiles from the pentapeptide hydrogel (2% *w/v*) were recorded. As shown in Figure 10, octreotide acetate release from the 2% *w/v* hydrogel reached 46.7 ± 2.4% within the first 2 h, followed by a more sustained release profile for 14 days (84.1 ± 3.4%). Approximately 66.1 ± 4.2% doxorubicin release from the 2% *w/v* hydrogel was achieved within the first 4 h, reaching 90.8 ± 2.0% within 14 days. The slightly slower release rate observed for the macromolecular octreotide acetate compared to the small hydrophilic doxorubicin hydrochloride from the peptide hydrogel, even though not statistically significant (*p* > 0.05), might be attributed to the higher molecular weight of the macromolecule, which delays drug diffusion within the hydrogel pores to the surrounding release medium [7]. Curcumin release from the 2% *w/v* peptide hydrogel reached only 20.2 ± 2.5% at 48 h, followed by a prolonged release profile for 14 days (81%). The more sustained release observed for curcumin, compared to octreotide acetate and doxorubicin hydrochloride, could be attributed to the drug’s hydrophobicity, which, as previously shown [51] and also verified here with the CLSM studies, favors interaction with the peptide nanofiber network, therefore leading to a more retarded release profile. Kinetic analysis of drug release from the pentapeptide hydrogel was performed and the release data of all drugs from the pentapeptide hydrogel correlated best to the Weibull model, as indicated by the respective correlation coefficient values reported in Table S5. The values of the exponent β, which is an indicator of the mechanism of drug transport, were calculated to be less than 0.75, suggesting a Fickian mechanism of drug diffusion though the hydrogel matrix [52]. It was only for curcumin release that β lay within the range of 0.75 < β < 1, indicating a combined mechanism of drug diffusion (Fickian) and swelling-controlled transport [53].

### 3.6. Cell Viability and Cell Death Assay 

**Figure 11:**
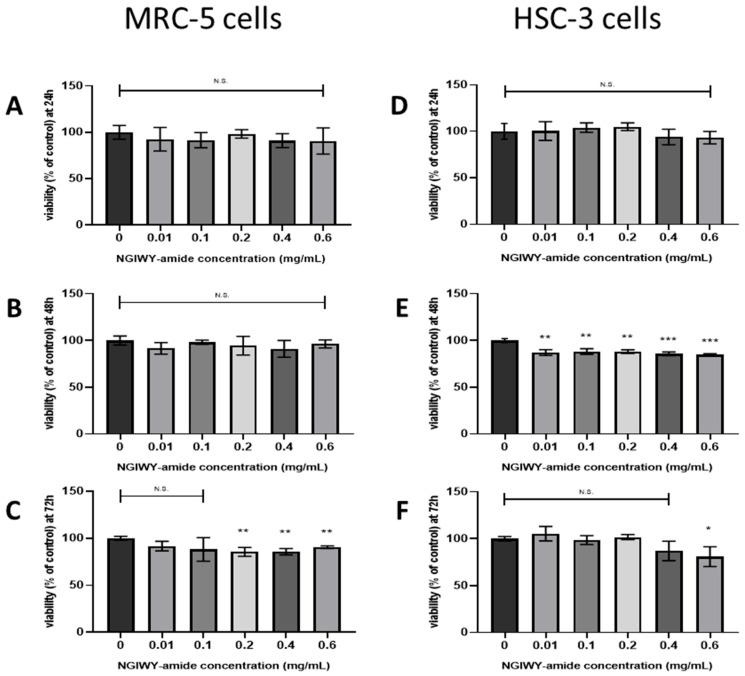
Cytotoxicity evaluation of the NGIWY-amide hydrogel. (**A**–**C**) Effect of NGIWY on MRC5 cell survival as assessed by CCK-8 analysis after treating with various concentrations of the NGIWY-amide pentapeptide hydrogel for (**A**) 24 h, (**B**) 48 h, and (**C**) 72 h. (**D**–**F**) Effect of NGIWY on HSC3 cells survival as assessed by CCK-8 analysis after treating with various dosages of NGIWY for (**D**) 24 h, (**E**) 48 h, and (**F**) 72 h. The data were analyzed using a *t*-test. Statistical significance was set at *p* < 0.05 (*: *p* < 0.05; **: *p* < 0.005; ***: *p* < 0.001).

In order to detect the cytotoxicity of NGIWY amide, MRC5 and HSC3 cells were allowed to grow in the presence of different concentrations of NGIWY for 24 h, 48 h, and 72 h. The results of cell viability by the Cell Counting Kit-8 (CCK-8) assay revealed that a low concentration of NGIWY (≤0.6 mg/mL) had no effect on the viability of MRC5 and HSC3 cells even after 72 h exposure (Figure 11). Additionally, no significant number of dead cells was observed to occur in cultures exposed to the treatments mentioned above as compared to control untreated ones (data not shown). Based on these results of the cell toxicity evaluation, NGIWY amide concentrations at ≤0.6 mg/mL were chosen for further experiments.

## 4. Discussion

In this study, we outlined and expanded on the properties of an ultra-short self-assembled cosmetic pentapeptide isolated from a marine invertebrate *Apostichopus japonicus* NGIWY-amide. Another cosmetic peptide, palmitoyl-KTTKS, has already been developed and commercialized as peptide hydrogel by Sederma Inc (France) under the brand name Matrixyl™. 

NGIWY-amide is a short marine pentapeptide with straightforward production with low-cost peptide synthesis incorporating Trp, an amino acid known for its intrinsic fluorescence. This inherent property enables the recording of its distribution and metabolism inside living cells without additional conjugation with fluorescent dye for labeling. However, aromatic stacking during assembly quenches tryptophan fluorescence [54]. Moreover, the pentapeptide displays excellent solubility in water, making it a promising candidate for several biocompatible applications. Tyrosine at the C-terminal site of the peptide enables the design and synthesis of phosphorylated analogs to form hydrogels in situ after their dephosphorylation in the presence of alkaline phosphatase, a common methodology in short-peptide hydrogels carrying Tyr [55,56,57]. NMR studies (Figure 2, Figure 3 and Figure 4) and MD simulations (Figure 5 and Figure 6) as well as spectroscopy and microscopy techniques were used to explore the robust pentapeptide hydrogel’s physicochemical parameters and structural properties (morphology, dimensions, etc.), identifying its potential as drug delivery system encapsulating curcumin, doxorubicin, and octreotide; drug molecules with different molecular weight; and aqueous solubility. Through π-π stacking interactions, various collagen like peptides assemble into higher hierarchical-ordered structures and mimic the fibril formation [58]. Li et al. reported that aromatic–aromatic interactions promote the pentapeptide transform from α-helix to β-sheet conformation, although isolated short peptides usually are unable to maintain their original secondary structures [59]. Self-assembled β-sheet peptides tend to form long fibrillar structures or tapes. TEM analysis further verified the findings of AFM studies, showing that the NGIWY-amide pentapeptide was highly filamentous, forming micrometer-long fibers with twisted morphology and a mean diameter of 5.6 ± 1.1 nm (Figure 9). Similar results were presented by Krysmann et al. for the KVLFF, an amyloid peptide fragment that forms fibrils itself in PBS (pH 7.4) [54]. In the same study, the long, stiff fibrils of the peptide hydrogel KLVFF exhibited a mean diameter of 9.68 ± 0.92 nm, and gelation took approximately 15 h for a 2.5% *w/v* sample, decreasing to 2 h for samples with concentrations of 3.0–4.0 *w/v*. However, NGIWY-amide needs less than 30 min to gel even at 1% *w/v*. It has been very recently shown that a short biomimetic eight amino acid residue peptide derived from squid-sucker ring-teeth proteins gels in water, forming a network of twisted nanofibers with a width of less than 10 nm and a periodicity of 80 nm along the fibers [50]. Tang et al. previously published [45] a class of rapidly assembling pentapeptides (KYFIL and analogs) for injectable delivery (RAPID) that form hydrogels with nanofiber structures. They also suggested that the deprotonated C-terminal site is necessary for the gelation of the peptide. This robust peptide hydrogel family is formed spontaneously at a low weight percentage (1.5 to 3% *w/v*), exhibiting a broad range of mechanical properties (50–17,000 Pa) that can be fine-tuned via small changes in concentration or pH. In addition, their rheological experiments indicated that Ile facilitates self-assembly. Further, NGIWY-amide, which incorporates the two aromatic amino acids at the C-terminal site, exhibited stiffness with a storage moduli G’ (G’max:160 kPa) (Figure 7). In peptide self-assembly, aromatic (Phe, Trp, Tyr) and aliphatic amino acids (Ala, Ile, Leu, Val) aggregate through π-π stacking and hydrophobic interactions, whereas the polar amino acids stabilize through electrostatic interactions or hydrogen bonds, depending on the charged residues. Predicting the molecular forces after peptide self-assembly is rather difficult without extra facts about the interactions of these forces.

One of the major drawbacks to the development of hydrogels as drug delivery systems is their inability to efficiently entrap and release poorly water-soluble drugs due to their own hydrophilic structure, and if it occurs, a burst release results in toxicity at the site of administration. Thus, NGIWY-amide delivered antitumor drugs of various molecular weight and hydrophobicity. The water-insoluble curcumin demonstrated a prolonged release profile for 14 days, in comparison to doxorubicin and octreotide acetate. Entrapment of the water-insoluble curcumin displayed a uniform distribution inside the gel with no observable aggregates. The high-MW (~1000) peptide octreotide acetate displayed a slightly slower release rate compared to the small molecule doxorubicin hydrochloride (~500), which might be attributed to the higher molecular weight of the macromolecule, which delays drug diffusion within the hydrogel pores to the surrounding release medium. The hydrochloric and acetate salts in doxorubicin and octreotide, respectively, disrupted the ionic interaction of the peptide hydrogel and enhanced the high release rate. On the opposite side, the lipophilic curcumin exhibited a more sustained release and favored the interaction with the peptide nanofiber network, therefore leading to a more retarded release. Short to ultra-short peptide hydrogels have already been used to deliver antitumor drugs such as doxorubicin [60,61,62] and curcumin [63,64,65]. In most cases, the synthesized peptides are based on the homodipeptide FF or its modified natural or unnatural amino acids, like napthylalanine. Gelation kinetics and the drug release over time are dependent on the peptide sequence. Recently, doxorubicin entrapped in a short nucleo-peptide hydrogel (Ade-FFF) released at a sustained rate (55% over 12 days) [66]. Other researchers used doxil instead of doxorubicin hydrochloride, so their results are not comparable, as doxil (the liposomal formula of doxorubicin) is expected to exhibit a different release rate [61]. Four different α,β-dehydrophenylalanine dipeptide nanotubes were used to encapsulate curcumin and investigate the management of malaria [64]. Eighty percent of the initial loaded curcumin concentration needed almost 90 h to be released from the nanosystem. Zhuo et al. reported a short rationally designed peptide hydrogel incorporating the Fmoc group an ocular implant for the prolonged release of antiproliferative drug 5-fluorouracil (5-FU); after 7 days, almost the total loaded 5-FU was released from the hydrogel [66].

Finally, the NGIWY-amide peptide hydrogel, a new candidate material for local drug delivery or other biomedical applications, was tested for its biocompatibility, a prerequisite criterion for clinical use. Biocompatibility of the pentapeptide hydrogel was assessed by CCK-8 analysis after treating both normal MRC5 and cancer HSC3 cells with various concentrations of the NGIWY-amide pentapeptide hydrogel (Figure 11). The results indicate that the growth of cells was unaffected, as no cytotoxicity was observed and the gel exhibited high biocompatibility.

## 5. Conclusions

In summary, we demonstrated for the first time the self-assembling properties of a natural known marine peptide (NGIWY-amide) isolated from sea cucumber *Apostichopus japonicus* and explored the robust hydrogel’s physicochemical parameters and its potential as a drug delivery system. NMR studies and MD simulations as well as spectroscopy and microscopy techniques were used to explore and identify the structural properties of the self-assembling peptide. Overall, the peptide hydrogel demonstrated (i) straightforward low-cost synthesis, (ii) gelation simplicity under physiological conditions, (iii) formation of a firm hydrogel, (iv) a simple encapsulation procedure, and (v) release of both hydrophobic and hydrophilic drug molecules and macromolecules in a controlled manner. NGIWY-amide peptide hydrogel addresses many of the drawbacks associated with existing peptide hydrogels for delivering therapeutics and is thus a promising candidate for further investigation as an in-situ gel-forming depot system for encapsulation and controlled drug delivery. Additionally, we observed hierarchical nanostructures (individuals nanofibers) that may offer new approaches for cell growth, drug delivery, regenerative medicine, tissue engineering, and 3D-printing-based tissue manufacturing even though bioelectronics. The field of bioinspired ultra-short self-assembling peptides is comparably new; novel discoveries and materials are expected to be explored and exploited for several future research directions and applications. We firmly believe that peptides from marine organisms like invertebrates and echinoderms pave new horizons in biomedicine.

## Data Availability

The raw/processed data required to reproduce these findings cannot be shared at this time, as the data also forms part of an ongoing study.

## Supplementary Materials

The following supporting information can be downloaded at: https://www.mdpi.com/article/10.3390/pharmaceutics14010133/s1, Table S1: ^1^H and ^13^C NMR chemical shifts of NGIWY in D_2_O, Table S2: ADME molecular properties of the pentapeptide, Table S3: MM2 job type, Table S4: Binding energy distribution of the NGIWY-amide with the transport protein, Table S5: Release kinetic parameters of octreotide acetate, doxorubicin hydrochloride, and curcumin from the pentapeptide hydrogel, Figure S1: Structure of the NGIWY-amide, Figure S2: HPLC profile of the synthesized peptide at 220 nm, Figure S3: Mass spectrum of the main peak at 10.79 min (PDF), Figure S4: Inverted vial of the hydrogel of the peptide.
