# Supplementary material for: NGIWY-Amide: A Bioinspired Ultrashort Self-Assembled Peptide Gelator for Local Drug Delivery Applications
Source: Pharmaceutics. 2022 Jan 6;14(1):133. doi: 10.3390/pharmaceutics14010133 (PMC8778326; doi:10.3390/pharmaceutics14010133)
Supplement: Supplementary file 1 [file pharmaceutics-14-00133-s001.zip › pharmaceutics-1528055-supplementary.pdf]

pharmaceutics

MDPI

Supplementary Materials: NGIWY-Amide: A Bioinspired Ultrashort Self-Assembled Peptide Gelator for Local Drug Delivery Applications

Nikoleta F. Theodoroula , Christina Karavasili , Manos C. Vlasiou , Alexandra Primikyri , Christia Nicolaou , Alexandra V. Chatzikonstantinou , Aikaterini-Theodora Chatzitaki , Christos Petrou , Nikolaos Bouropoulos , Constantinos K. Zacharis , Eleftheria Galatou, Yiannis Sarigiannis , Dimitrios G. Fatouros and Ioannis S. Vizirianakis

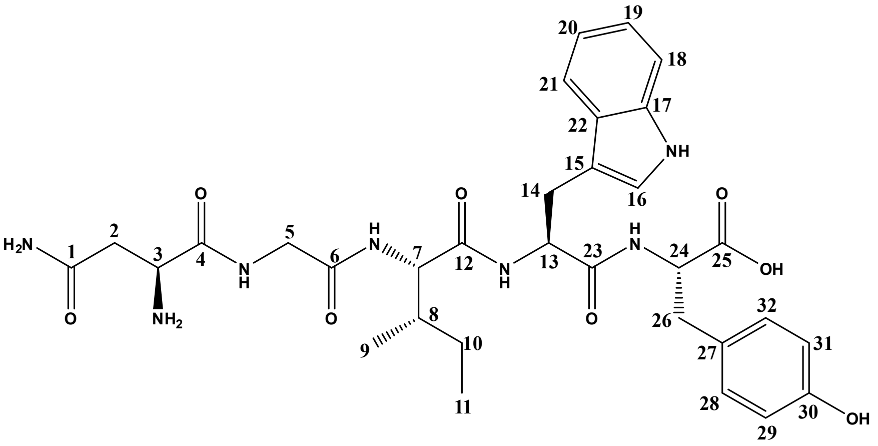

Table S1. ^{1}H and ^{13}C NMR chemical shifts (δ, ppm) of NGIWY in D_2O.

H	δ H	C	δ C	Long range connectivities
**9**	0,56 (d, 3H)	**9**	14,4	24.5 (C10), 36 (C8), 58.4 (C7)
**11**	0,71 (t, 3H)	**11**	10,1	24.5 (C10), 35.9 (C8)
**10α**	0,96 (m, 1H)	**10**	24,5	10 (C11), 14.4 (C9), 36.02 (C8)
**10β**	1,20 (m, 1H)			
**8**	1,60 (m, 1H)	**8**	36,0	
**26α**	2,64 (d, 1H)	**26**	36,1	50.3 (C3), 54.5 (C24), 128.1 (C27), 130.3 (C28,32), 173.5 (C25)
**26β, 2α+β**	2,81 (m, 3H)	**2**	36,0	54.5 (C24), 128.1 (C27), 130.3 (C28,32), 175.1 (C1), 173.5 (C25)

**14α+β**	3,11 (d, 2H)	**14**	27,1	54.3 (C13), 108.8 (C15), 124.6 (C16), 126.27 (C22), 172.56 (C23)
**5**	3,89 (dd, 2H)	**5**	42,3	
**7**	3,99 (d, 1H)	**7**	58,4	14.4 (C11), 24.4 (C10), 36 (C8), 172.79 (C12)
**3**	4,18 (quin, 1H)	**3**	50,2	
**24**	4,31 (q, 1H)	**24**	54,5	
**13**	4,57 (q, 1H)	**13**	54,3	
**29,28**	6,70 (d, 2H)	**29, 28**	115,3	115.3 (C29), 128 (C27), 154.3 (C30)
**31,32**	6,92 (d, 2H)	**31, 32**	115,3	36.19 (C26), 115.2 (C32), 130.4 (C31), 154.4 (C30)
**19**	7,09 (t, 1H)	**19**	119,4	111.7 (C18), 126.6 (C17)
**16**	7,12 (s, 1H)	**16**	124,5	108.6 (C15), 126.6 (C17), 136.1 (C22)
**20**	7,17 (t, 1H)	**20**	121,9	118.3 (C21), 135.9 (C22)
**18**	7,42 (d, 1H)	**18**	111,8	119.4 (C19), 126.5 (C17)
**21**	7,55 (d, 1H)	**21**	118,2	108.4 (C15), 122 (C20), 136.1 (C22)

Table S2. ADME molecular properties of the pentapeptide.

**Water Solubility**	
Log *S* (ESOL)	-2.75
Solubility	1.20e+00 mg/ml ; 1.76e-03 mol/l
Class	Soluble
Log *S* (Ali)	-5.35
Solubility	3.01e-03 mg/ml ; 4.44e-06 mol/l
Class	Moderately soluble

Log *S* (SILICOS-IT)	-6.63
Solubility	1.61e-04 mg/ml ; 2.37e-07 mol/l
Class	Poorly soluble
**Pharmacokinetics**	
GI absorption	Low
BBB permeant	No
P-gp substrate	Yes
CYP1A2 inhibitor	No
CYP2C19 inhibitor	No
CYP2C9 inhibitor	No
CYP2D6 inhibitor	No
CYP3A4 inhibitor	No
Log K_p (skin permeation)	-10.52 cm/s
**Lipophilicity**	
Log $P_{o/w}$ (iLOGP)	1.02
Log $P_{o/w}$ (XLOGP3)	-0.11
Log $P_{o/w}$ (WLOGP)	-1.47
Log $P_{o/w}$ (MLOGP)	-2.58
Log $P_{o/w}$ (SILICOS-IT)	1.68
Consensus Log $P_{o/w}$	-0.29
**Drug-likeness**	
Lipinski	No; 3 violations: MW>500, NorO>10, NHorOH>5
Ghose	No; 4 violations: MW>480, WLOGP<-0.4, MR>130, #atoms>70
Veber	No; 2 violations: Rotors>10, TPSA>140

Egan	No; 1 violation: TPSA>131.6
Muegge	No; 4 violations: MW>600, TPSA>150, Rotors>15, H-don>5
Bioavailability Score	0.17
**Medicinal Chemistry**	
PAINS	0 alert
Brenk	1 alert: diketo_group
Leadlikeness	No; 2 violations: MW>350, Rotors>7
Synthetic accessibility	5.55

Table S3. MM2 Job Type.

**Menu Name:**	Molecular Dynamics
**Current Options Tab**	4
**Display Iterations Job Type**	Molecular Dynamics
**Menu Name**	Molecular Dynamics
**Current Options Tab**	4
**Display Iterations Job Type**	Molecular Dynamics
**Minimum RMS Gradient**	0.00000

**Step Interval:**	2.00000
**Frame Interval:**	10
**Has Terminate Dynamics Terminate Steps**	10000
**Is Heating**	
**Heating Rate:**	1.00000
**Target Temperature**	300
**Is Saving Step Data**	
**Step Data File:**	C:\Users\chemo\Desktop\NGIWY\

Table S4. Binding energy distribution of the NGIWY-amide with the transport protein.

**Total Energy**	**VDW**	**HBond**	**Elec**
**-87.1426 Kcal/mol**	**-43.709 Kcal/mol**	**-43.4337 Kcal/mol**	**0 Kcal/mol**

**Amino Acid Residue**	**Energy (Kcal/mol)**	**Amino Acid Residue**	**Energy (Kcal/mol)**	**Amino Acid Residue**	**Energy (Kcal/mol)**	**Amino Acid Residue**	**Energy (Kcal/mol)**
H-M-GLY-345	-2.39728	V-S-PRO-344	-0.537867	V-M-SER-352	-6.73952	V-M-NAG-2	-1.258
H-S-ASN-350	-3.5	V-M-GLY-345	-1.0201	V-S-SER-352	-0.775445	V-S-NAG-2	-7.26528
H-M-GLU-351	-0.70163	V-M-PHE-346	-0.476752	V-M-LEU-353	-10.0653	V-M-FUC-3	-1.62757
H-M-SER-352	-3.5	V-M-SER-347	-0.00020353	V-S-LEU-353	-5.52406	V-M-FUC-3	0.444912
H-M-LEU-353	-3.5	V-M-ASN-350	-1.3596	V-M-ILE-354	-2.87016		
H-M-NAG-2	-14.2554	V-S-ASN-350	-9.34123	V-S-ILE-354	-1.05764		
H-S-NAG-2	-15.5793	V-M-GLU-351	-3.36612	V-M-GLU-358	-0.140485		
V-M-PRO-344	-1.40608	V-S-GLU-351	-2.07376	V-S-GLU-358	-7.37926		

Table S5. Release kinetic parameters of octreotide acetate, doxorubicin hydrochloride and curcumin from the pentapeptide hydrogel.

	Zero order		First order		Korshmeyer-Peppas			Higuchi		Hixson-Crowell		Weibull		
**Formulation**	R^2	k_0	R^2	k_1	R^2	K	n	R^2	K_H	R^2	K_{HC}	R^2	α	β
2% w/v NGWIY-OCT	-2.202	0.344	0.499	0.312	0.904	37.593	0.318	-0.624	6.250	-0.783	0.004	0.966	1.619	0.206
2% w/v NGWIY-DOX	-1.763	0.376	0.841	0.365	0.983	38.412	0.396	-0.385	6.839	-0.479	0.005	0.984	1.497	0.256
2% w/v NGWIY-CUR	0.815	0.291	0.950	0.006	0.959	4.824	0.498	0.982	4.667	0.927	0.002	0.990	105.302	0.899

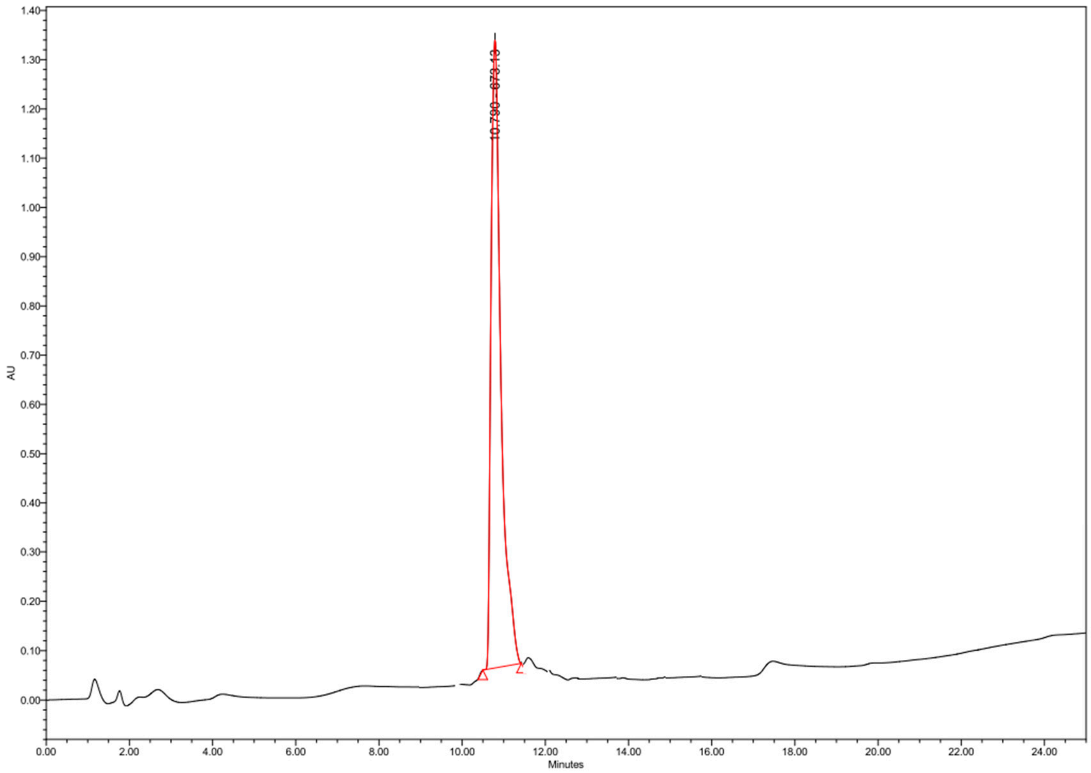

Figure S1. HPLC profile of the synthesized peptide at 220 nm.

pharmaceutics

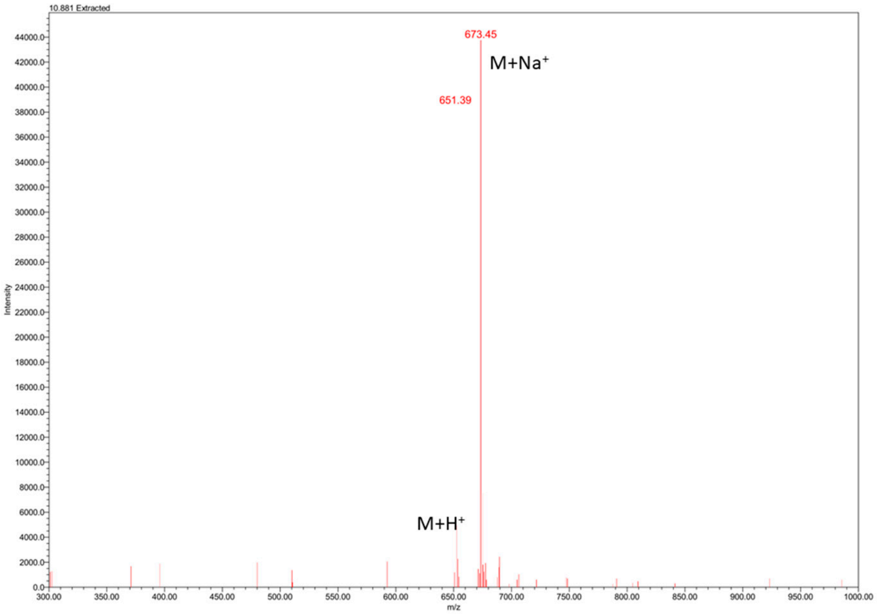

Figure S2. Mass spectrum of the main peak at 10.79 min.

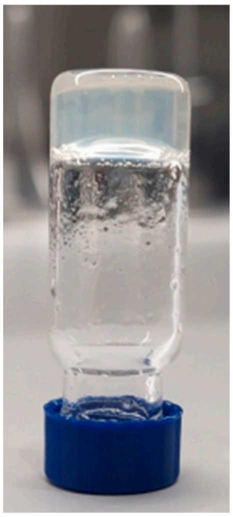

Figure S3. Inverted vial of the hydrogel of the peptide NGIWY

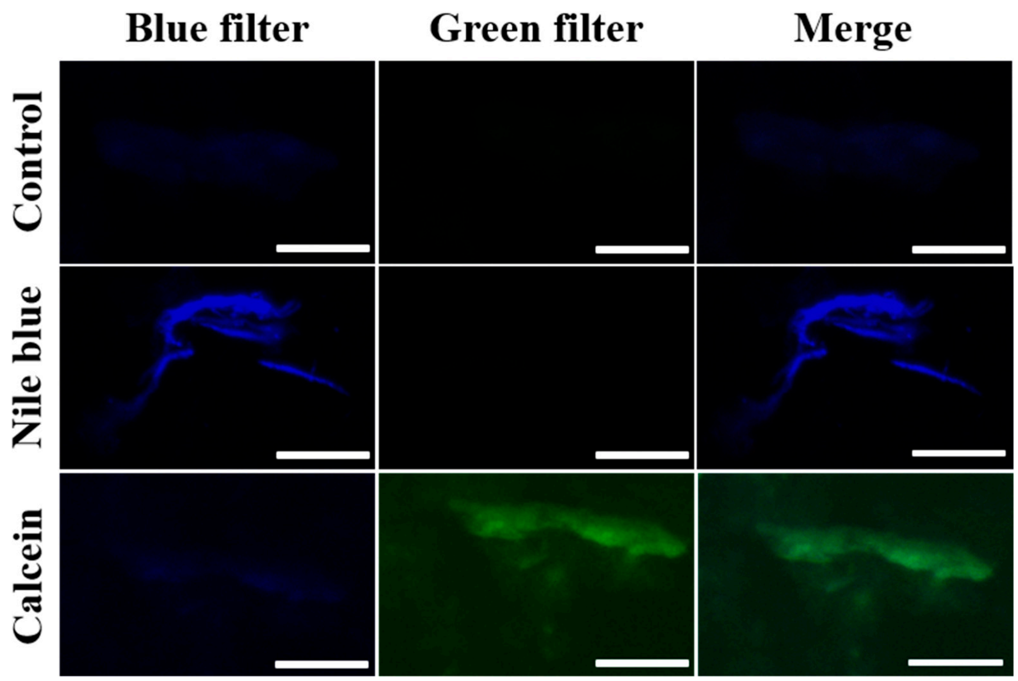

Figure S4. Confocal images (63x objective) of a lipophilic (Nile blue) and a hydrophilic (calcein) fluorescent dye distribution within the pentapeptide hydrogel. Scale bar: 10 µm.
